# Ecological Risk Assessment of Heavy Metals along Three Main Drains in Nile Delta and Potential Phytoremediation by Macrophyte Plants

**DOI:** 10.3390/plants9070910

**Published:** 2020-07-18

**Authors:** Yasser A. El-Amier, Giuliano Bonanomi, Saud L. Al-Rowaily, Ahmed M. Abd-ElGawad

**Affiliations:** 1 Department of Botany, Faculty of Science, Mansoura University, Mansoura 35516, Egypt; yasran@mans.edu.eg; 2 Department of Agriculture, University of Naples Federico II, Portici, 80055 Naples, Italy; giuliano.bonanomi@unina.it; 3 Plant Production Department, College of Food & Agriculture Sciences, King Saud University, P.O. Box 2460, Riyadh 11451, Saudi Arabia; srowaily@ksu.edu.sa

**Keywords:** phytoremediation, *Phragmites australis*, *Typha domingensis*, bioaccumulation, heavy metals, pollution indices

## Abstract

The use of drainage water in the irrigation of agroecosystem is associated with environmental hazards, and can pose threats to human health. Nine heavy metals (Fe, Mn, Zn, Cu, Co, Cr, Ni, Cd and Pb) along three main drains in the middle Nile Delta were measures in the sediments, roots and shoots of three common macrophytes (*Echinochloa stagnina*, *Phragmites australis* and *Typha domingensis*). The physicochemical characteristics, as well as the enrichment factor (Ef), contamination factor (Cf), geoaccumulation index (Igeo), ecological risk factor (Er), degree of contamination (Dc) and potential ecological risk index (PERI), were determined for sediment. The metal bioaccumulation factor (BAF) and translocation factor (TF) were assessed for plants. Data revealed high contents of Cr, Zn and Cd in the upstream of the drains, while Mn, Cu and Ni were recorded in high concentrations in the downstream. Mn, Cr, Co, Cu, Ni and Zn were recorded to be within EU (2002), CSQGD (2007) and US EPA (1999) limits, while Cd and Pb showed high a ecological risk index. This high concentration of pollutants could be attributed to unremitting industrial activities, which can bioaccumulate in the food chains and cause serious problems for humans. The root of *P. australis* showed the effective accumulation of most of the elements, while *T. domingensis* revealed the highest accumulation of Pb. However, the highest BAF shoot value was found in *T. domingensis* for most of the heavy metals, except for Fe and Zn in *P. australis* and Mn in *E. stagnina*. Thus, *P. australis* could be used as a potential phytoextractor of these hazardous metals, as an eco-friendly and cost-efficient method for remediation of the polluted drains. Further, *T. domingensis* could be integrated as a hyperaccumulator of Pb. Strict laws and regulations must be taken into consideration by the policymaker against unmanaged industrial activities, particularly near the water streams in the Nile Delta.

## 1. Introduction

In arid and semi-arid regions, the insufficiency of freshwater resources is the main factor limiting the expansion of the cultivated land to meet the food demands required by the progressively increasing population [1]. Nowadays, nearly 40% of the world’s food supply is grown under irrigation [2]. Therefore, to overcome the shortage of food demands and to reach a satisfactory level of food production, the utilization of other water resources besides the fresh ones is now urgent. Development of the economy is highly influenced by water availability, seasonality, and quality as well [3].

Pollution is one of the most serious factors that influences the vulnerability of water. The main sources of the pollution are untreated industrial effluents, municipal wastewater, runoff from chemical fertilizers and pesticides from the agriculture sector [4]. Inorganic chemicals have been considered a major threat to aquatic ecosystems and, in consequence, to human health. Usually, the agricultural drainage water receives several contaminants that originate from industrial, agricultural and municipal activities. All these activities contaminate the water bodies and sediments with huge quantities of inorganic anions and heavy metals [5]. The municipal sewage sludge, as an end product of domestic wastewater, contains organic pollutants as well as heavy metals, which integrated into the agriculture sector and thereby into the food chain, which affects human health [6,7].

In Egypt, particularly in the Nile Delta, the rate of population growth imposes high stress on the agriculture sector in order to meet food demands for human [8]. As a result, in many parts of the Nile Delta, farmers use wastewater for irrigation due to the limited resources of water required for agricultural expansion in this over-exhausted agricultural area. The use of wastewater for irrigation leads to an increase in the level of heavy metals in agricultural soils [6]. Therefore, agroecosystem practices affect food quality and safety, thus having a great impact on human health [9].

Heavy metals include 38 elements, and some of these metals, such as iron, copper, manganese and zinc, are useful as micro-nutrients for more developed plants and humans when taken up in acceptable amounts. However, when these elements exceed the limits in the plant cell, they become poisonous and inhibit the normal growth of plants, reducing crop yield and causing health problems [10]. Heavy metals are not biodegradable, and persist for a long time in the soil [11]. The risk of human exposure to heavy metals through the food chain increases when the crops are grown in contaminated soil. Today, mutagenesis and carcinogenesis are correlated with the chronic exposure of humans to some of the heavy metals [12]. The use of herbicides, fertilizers, livestock manure, and wastewater for irrigation in agricultural soils are the primary sources of heavy metals [9,13]. Sediments at the bottom of the water streams play an important role in the pollution scheme of the water body. Thus, the concentration of heavy metals in the sediment reflects the toxicity level in the water body [14].

Macrophytes are successful in absorbing contaminants from soils and waters; therefore, they play an important role in sequestering large quantities of nutrients and metals from the ecosystem, by storing them in the roots and/or shoots [15]. The extent of absorption mostly depends on plant species, as well as the bioavailability and ionic potential of the element concerned [16]. Phytoextraction is one of the phytoremediation processes, whereby plants can uptake metals from soil, and it is thereby used to remove heavy metals from the contaminated soil [17]. In this regard, macrophytes are considered as bioindicators for heavy metals [18,19]. The phytoremediation of heavy metals by aquatic macrophytes is considered an eco-sustainable, green-driven, cost-effective and passive technique for environmental management [17,19,20,21]. Several macrophyte plants have been reported as phytoremediators of various heavy metals, such as *Phragmites australis* (Cav.) Trin. ex Steud. [22,23,24,25,26,27], *Echinochloa stagnina* (Retz.) P. Beauv. [27], *Typha domingensis* (Pers.) Poir. ex Steud. [22,28], *T. latifolia* L. [29], *Potamogeton pectinatus* L. and *P. malaianus* Miq. [30].

In Egypt, the coastal lakes of the Nile Delta (Mariut, Idku, Burullus and Manzala) and other aquatic ecosystems are directly and/or indirectly affected by various anthropogenic activities developing along the watersheds, which receive inputs of polluted water that contain organic and inorganic compounds from point and non-point sources [31]. The gradual increase of these different pollutants is also associated with the changes in the composition of aquatic vegetation over time. The drainage system of the Nile Delta is extensive, serving 64% of the total 29,600 km^2^ of agricultural land in Egypt, which comprises many major drains. The usage of drainage water in irrigation for the agrosystem must be controlled due to the high contents of pollutants, which in turn may cause hazards to the soil, crop yield and quality, and human health. In the present study, our aims were as follows: (i) to evaluate the pollution status, by nine heavy metals (Fe, Mn, Zn, Cu, Co, Cr, Ni, Cd and Pb), in three main drains along the middle Nile Delta, which discharge into Burullus Lake; (ii) to assess the environmental risk along these drains using different pollution indices; and (iii) to determine the potentiality of three common and perennial emergent hydrophytes that inhabit the banks of these drains, for use as phytoextractors of the heavy metals considered in this study.

## 2. Results and Discussion

### 2.1. Sediment Analysis

#### 2.1.1. Physiochemical Parameters

The environmental mobility and availability of heavy metals in sediments are controlled by their physicochemical properties, including the total metal content, pH, Electrical conductivity (EC), organic matter (OM), grain size, and calcium carbonate and oxides contents [10,32,33,34]. The present data show a significant difference (*p* ≤ 0.05) in the EC, OM, sand and silt values between the positions (upstream, middlestream and downstream) of the three studied drains (Table 1). However, no significant differences were observed in pH, CaCO_3_ or and clay values. The mean sediment pH ranged from 7.84 in the downstream to 8.00 in the upstream, i.e., strongly alkaline. The differences in pH among locations are not significant. Kelepertzis and Stathopoulou [32] reported that soil pH strongly affects the solubility and bioavailability of metals. For example, the dissolution of heavy metals in soil solution increases with a decrease in pH [35]. In contrast, with high soil salinity, heavy metals form a complex with chloride ions, and consequently the bioavailability of the metals often decreases [36]. The EC ranged from 1.98 dS/m^−1^ in the upstream to 3.50 dS/m in the downstream, due to the seepage of saltwater from the Mediterranean Sea. It is worth mentioning here that the Nile Delta region faces a serious problem of salinity and sodicity [8]. Based on the World Reference Base for soil resources, most of the Egyptian soil groups are alluvial soils. According to soil groups/land cover, the Nile Delta soils range from calcaric fluvisols to gleyic solonchaks [37]. Furthermore, the soil becomes sandier as one moves toward the downstream (Table 1).

On the other hand, the OM in the present study ranged from 1.40% in the upstream to 2.59% in the downstream. The role of OM in metal availability has been extensively investigated [33,34,38]. It was found that increasing the content of OM led to an increased mobility and availability of heavy metals to plant roots. Additionally, the CaCO_3_ content ranged from 2.38% in the middlestream to 2.73% in the downstream. Dube et al. [39] reported that organic mineral particles have a great influence on the texture of the soil. In all samples, sediments were found to be sandy in texture, with sand contents ranging from 76.93% to 88.92%. Variations in soil texture were dependent on the geological nature of the Nile Delta.

#### 2.1.2. Heavy Metal Distribution in Sediments

Heavy metals are metals that occur naturally in the Earth’s crust in a specific concentration. Anthropogenic activities lead to increases in the heavy metal levels in the ecosystems, which consequently increases the pollution and human health risk [10,12]. In the present study, we identified significant variations in the concentrations of Cr, Cd and Mn among the different streams (upstream, middlestream and downstream), with the highest concentration recorded at the downstream (sites 3, 6 and 9) for most metals. However, no significant variations were recorded for Fe, Pb, Zn, Cu, Ni or Co (Table 2).

Our results showed that the most abundant element is iron, with a concentration in the sediments that varied from 46301.33 mg/kg in the upstream to 51412.33 mg/kg in the downstream (Table 2). As expected, Fe is a common element in any environment, and is ranked the third most abundant element in the Earth’s crust after oxygen and silicon [40,41]. The Fe distribution in the water stream is influenced by anthropogenic activities (steel industry, sewage, etc.); in addition, iron sulfate is also used in the fertilizer and herbicide industries [42]. In agreement with Farhat [40], who stated that iron and manganese are closely associated in a geochemical cycle, the concentration of manganese came after that of iron.

The mean level of manganese ranged from 788.30 mg/kg in the middlestream to 972.27 mg/kg in the downstream (Table 2). Manganese is an essential metal in plant growth, but excess levels of Mn in the soil can also hamper plant growth due to its competing for transport and metabolism with other cationic elements. In the present study, the Mn level fell within the range of tolerance for most plants [43]. However, the availability of Mn to plants is correlated with different factors, including its forms, dynamics and pH [44]. The elevated concentration of Mn in the sediments could be attributed to the runoff of sewage from different villages along the drains, and the release of organic matter and fertilizers from the agriculture sector [7].

The highest value of Pb (754.59 mg/kg) was recorded in the middlestream of the studied drains, while the lowest value (541.25 mg/kg) was detected in the downstream. Numerous studies have identified the main source of Pb as anthropogenic output, such as automobile exhausts and car batteries, industrial effluents, sewage sludge, fertilizers, and pesticide application [7,45].

**Figure 1:**
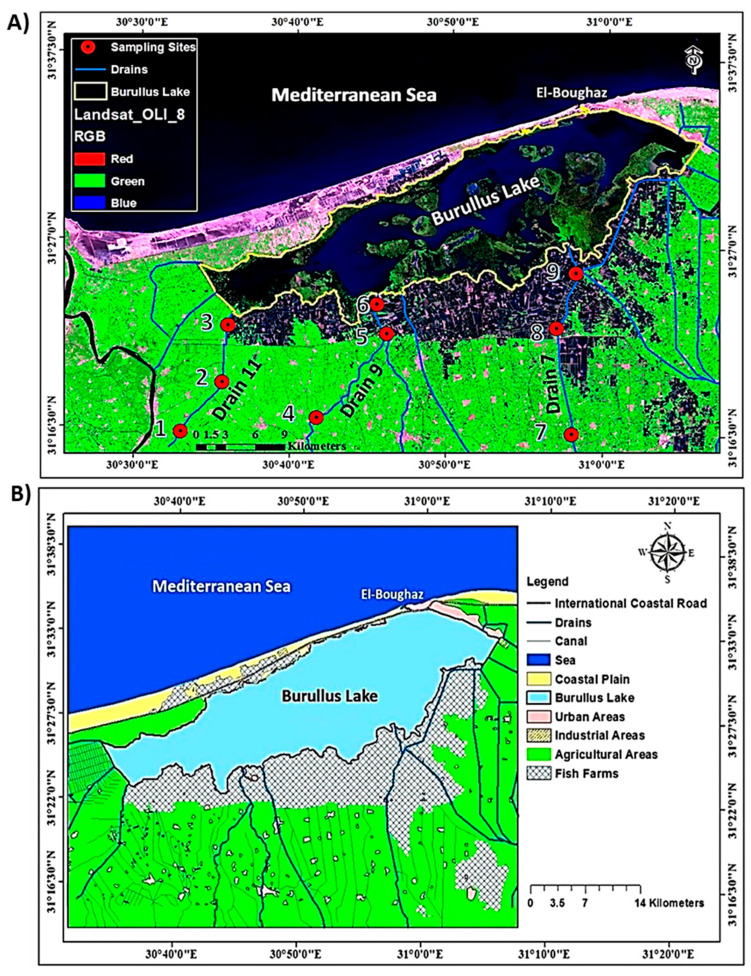
Location map showing studied sites in the middle Nile Delta. (**A**) the location of the studied sites (1–9) along the three drains. (**B**) land use map of the middle Nile Delta region with various drains discharged into Burullus Lake.

The distribution of Cr, Zn and Cd indicated an incremental increase in the concentrations from upstream to downstream, with ranges of 48.77–443.50 mg/kg, 34.13–42.53 mg/kg and 2.65–41.80 mg/kg, respectively (Table 2). The highest concentrations of these three heavy metals were recorded upstream of the three drains (sites 1, 4 and 7), while the lowest levels were further north, in the downstream areas (sites 3, 6 and 9). The cadmium background levels were low, so any elevated Cd concentrations can be associated with runoff from agricultural land where fertilizers, herbicides and pesticides are used. Environmental contamination with Cr in the sampled sites could be ascribed to agricultural activity, particularly the use of ammonium fertilizers, as well as industrial processes such as chromium plating, metal finishing, leather tanning and paint production [46,47]. The highest Zn concentration is mainly associated with the content of OM and the extractable carbonate fraction [48]. These sources of pollution were concentrated in the middle region of the Nile Delta, as shown in the land use map (Figure 1B).

In contrast, the highest concentrations of Cu, Ni and Co were observed downstream of the three drains (sites 3, 6 and 9), while the lowest levels were northward, in the upstream areas (sites 1, 4 and 7). The distributions of Cu, Ni and Co showed trends of consistency with the sites of drains, with ranges of 8.42–12.24 mg/kg, 3.65–6.24 mg/kg and 31.40–32.43 mg/kg, respectively. Copper does not seem to be homogeneously distributed at all the sites. Usually, Cu contamination results from various anthropogenic activities, such as fungicidal treatments, sewage sludge, mining, and particles from car brakes [49]. Most of the Cu, Co and Ni metal measurements are associated with organic compounds, carbonates and Fe/Mn oxides [48]. Co presents a low mobility and strong adsorption in the soils, although its mobility increases in moist, acidic soils [38]. The downstream region of the middle Nile Delta is mainly fish farms (Figure 1B), which received considerable quantities of sewage effluent and agricultural runoff. These practices have led to the increasing of the content of heavy metals in this region [8]. The high content of Ni in the downstream area could be attributed to its low mobility [32].

The mean concentrations of the measured heavy metals in the investigated drains were found to be in the following order: Fe > Mn > Pb > Cr > Zn > Co > Cd > Cu > Ni. The highest concentrations of Mn, Cr, Zn, Cd and Co were detected upstream, while the highest concentrations of Fe, Ni and Cu were downstream, and Pb in the middlestream area (Table 2). To assess the potential adverse biological effects and the sediment toxicity of the sediments, a comparison with the EU [50], CSQGD [51] and US EPA [52] for the present results was done. According to US EPA [52], only the average value of Mn was in the higher range, while the average values of Pb, Cd, Cr and Co were in the higher range for EU [50], CSQGD [51] and US EPA [52]. By comparing the average values of Cu, Ni and Zn with EU [50], CSQGD [51] and US EPA [52], it was clear that concentrations of all these metals were in the lower range, as seen in Table 2.

#### 2.1.3. Correlation between Sediment Parameters and Heavy Metals

The Pearson correlation coefficient (r) analysis of various parameters of the sediments and heavy metals revealed that Cr was positively correlated (*p* ≤ 0.05) with silt, Cd and clay (r = 0.94, 0.89 and 0.83, respectively), while it was negatively correlated with EC, sand, OM, Cu and Ni, with r values of −0.95, −0.93, −0.83, −0.71 and −0.71, respectively (Table S1). The positive correlation of Cr with clay and silt contents was reported by Shabankareh et al. [34]. In contrast with our result, Kierczak et al. [38] and Shabankareh et al. [34] showed that Cr is positively correlated with OM.

Cadmium showed a positive significant linear correlation with silt (r = 0.82) and was negatively correlated with EC, OM and sand content, with which it attained Pearson correlation coefficient values of −0.85, −0.84 and −0.71, respectively (Table S1). In agreement with our results, Spurgeon et al. [53] showed that Cd and Pb are not correlated with the pH and OM.

Salinity was positively correlated with OM and sand fraction (r = 0.78 and 0.90). Ni showed a positive correlation with OM (Table S1). Kelepertzis and Stathopoulou [32] reported that Ni availability is correlated with the organic carbon content in the soil.

Although pH is considered as a key parameter controlling metal availability [38], the present data did not show any significant correlation between the pH and studied heavy metals. In this context, Dragović et al. [54] showed that Cd, Cr, Cu, Mn, Ni, Pb and Zn have positive correlations with the OM and clay content, but not with pH.

### 2.2. Contamination Assessment of Heavy Metals in Sediment

Pollution indices are used as tools for assessing the degree of contamination in the environment, and the ecological risk index has been proposed to describe the translocation of heavy metals in soils and plants [55]. In addition, these indices are used to determine the ecotoxicological effects and health risks of the intake of contaminated food crops. Therefore, the remediation processes and efforts should be directed towards reducing metal concentrations in the soil, so as to minimize the health risks [56].

#### 2.2.1. Enrichment Factor (EF)

**Figure 2:**
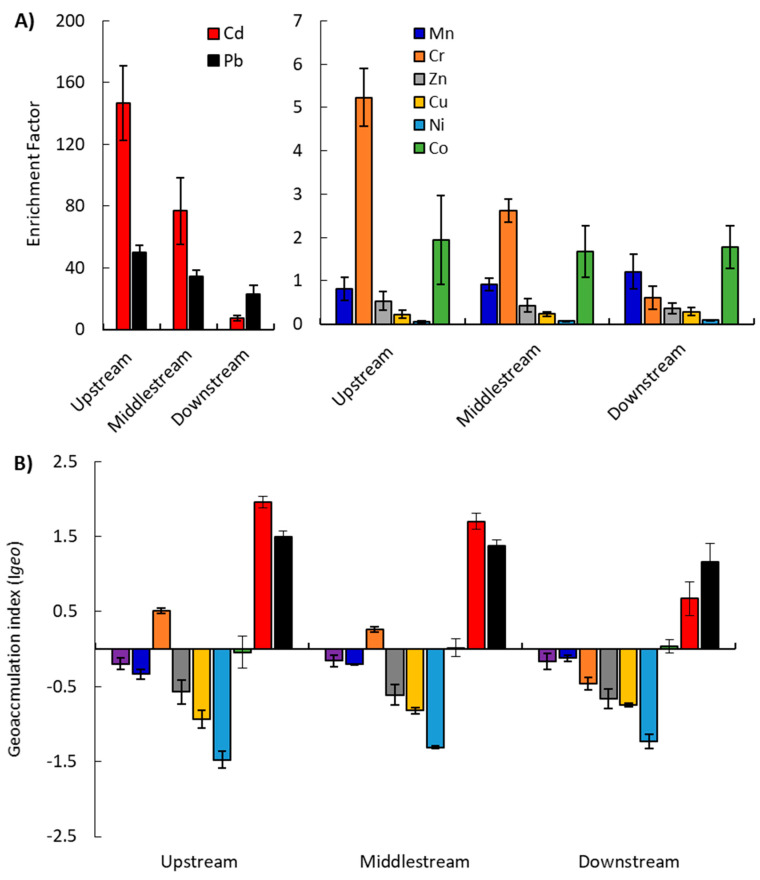
The enrichment factor **(A)** and geo-accumulation index **(B)** of the heavy metals in the sediment samples from different locations (up-, middle- and downstream) of the studied drains in the Nile Delta.

The EF was employed to assess the impacts of anthropogenic contamination on the concentration of heavy metals in sediments. In this investigation, iron was chosen as the reference element for EF values determination [57]. The measured ranges of EF for Mn, Cu and Ni were 0.81–1.21, 0.23–0.29 and 0.06–0.09, downstream to upstream, respectively. The EF for Zn ranged from 0.37 downstream to 0.54 upstream, while that for Co ranged from 1.68 in the middlestream area to 1.94 upstream (i.e., depletion or no enrichment). The enrichment factor for the Cr metal ranged between 0.61 downstream and 5.23 upstream (i.e., ranged from depletion to moderate enrichment). The calculated ranges of EF for Cd and Pb were 7.42–146.60 and 22.98–49.80, downstream and upstream, respectively (i.e., they ranged from severe, to very severe, to extremely severe enrichment). The investigated metals were ranked for their EF values in a descending order, as follows: Cd > Pb > Co > Cr > Mn > Zn > Cu > Ni (Figure 2A). According to Liu et al. [57], an EF within the range 0.05–1.50 suggests that the heavy metal concentration comes entirely from crustal materials and natural processes, while an EF *>* 1.50 was derived from non-crustal materials, like point and non-point anthropogenic sources. On this basis, Cd, Cr and Pb were likely introduced to the Burullus drains from anthropogenic origins (Figure 2A). The bioavailability and toxicity of the metals in the sediment samples depend not only on the concentrations of metals, but also on their available chemical forms [58].

#### 2.2.2. Geo-accumulation Index (Igeo)

The Igeo is considered to be the most accurate and used index for assessing heavy metal accumulations in aquatic sediments. The Igeo was calculated with respect to the geochemical background value of the element in the average shale [59]. The present Igeo results for the investigated metals indicated that the studied drain’s sediments can be classified as class 0 (Igeo < 1; i.e., uncontaminated; Fe, Mn, Zn, Ni and Cu). Meanwhile, the Igeo value for Cr and Co fall into two classes, namely, class 0 down- and upstream, respectively, and class 1 at other sites of the studied area (i.e., uncontaminated to moderately contaminated). These classes indicate varying sediment quality and local contamination. The Igeo values for Cd and Pb fall in class 2 (1 < Igeo < 2) in most sites of the drains, indicating moderate to heavy contamination, except Cd, which falls in class 0 downstream (i.e., uncontaminated). Therefore, according to Müller [60], the drains of the Burullus Lake are uncontaminated with Fe, Mn, Zn, Ni and Cu, while moderately to heavily contaminated with Cd and Pb, with an order of Cd > Pb > Cr > Co > Ni > Cu > Zn > Mn > Fe (Figure 2B).

#### 2.2.3. Contamination Factor (Cf) and Contamination Degree (CD)

**Figure 3:**
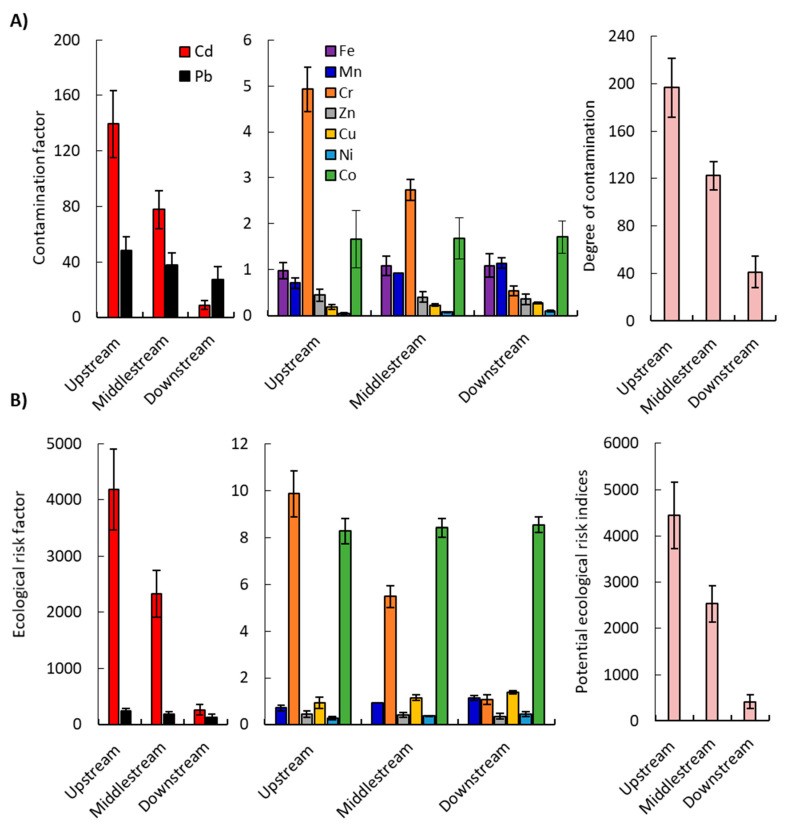
(**A**) The contamination factors and degree of contamination of heavy metals in the sediment samples from different locations (up-, middle- and downstream) of the studied drains in the Nile Delta. (**B**) Ecological risk factor and potential ecological risk index.

The contamination factor (Cf) enables the assessment of sediment contamination, taking into account the ratio of metal concentration (C*_sample_*) in the sediment and metal concentration in the unpolluted sediment (C*_ref_*). The content of metals in the Earth’s crust is used as a reference value for the evaluation of the soil’s heavy metal contamination [61]. The present results indicate that the values of Cf for Mn, Zn, Cu and Ni are < 1 (low contamination) in all sites, except for Mn downstream (Cf = 1.14). On the other hand, Fe and Co showed moderate contamination (1 ≤ CF ≤ 3) at all sites, except Fe upstream (Cf = 0.98). Chromium showed a Cf ranging between low contamination (Cf = 0.54) downstream, moderate contamination (Cf = 2.73) in the middlestream, and considerable contamination (Cf = 4.93) upstream. Meanwhile, the Cf values of Cd and Pb displayed remarkable variation amongst the studied sites, with the highest concentrations (139.32 and 48.40) recorded upstream (i.e., very high contamination) (Figure 3A).

Furthermore, the average degree of contamination (D*_C_*) indicated that most of the sites attained D*_C_* > 28 (i.e., very high degree of contamination), with values of 41.09, 122.50 and 196.68 in the down-, middle- and upstream areas, respectively. According to Hakanson [61] and Caeiro et al. [55], the results obtained indicate serious anthropogenic pollution. Contaminants are transported from upstream (i.e., agricultural land, sewage, and tributaries) and subsequently deposited downstream.

From the above results of the pollution indices, we have observed that Igeo showed the same tendency as E*f* and Cf, indicating that the drains discharging into Burullus Lake are mostly uncontaminated (i.e., of natural origin), except for the moderate to heavy Cd and Pb contamination (i.e., anthropogenic origin).

#### 2.2.4. Ecological Risk Assessment

The ecological risk index (E*r*) of single heavy metals in the sediments was described using the potential ecological risk index (PERI). The PERI was applied to assess the ecological sensitivity of heavy metal pollution in the stream’s sediments, according to the toxicity of heavy metals and the responses of the environment [61]. The results of the evaluation of E*r* and PERI are shown in Figure 3B. The E*r* of heavy metals in the sediments of the drains can be ranked as follows: Cd > Pb > Co ≈ Cr > Cu > Mn > Zn > Ni (Figure 3B). The mean values of the E*r* index for Mn, Cr, Co, Cu, Ni and Zn were lower than 40 (Er < 40; i.e., low ecological risk). The mean E*r* values of Cd and Pb were 264.79–4179.67 and 135.31–241.99, downstream and upstream, respectively (i.e., ranged from considerable (80 < Er < 160) to high (160 < Er < 320) to very high (Er ≥ 320) ecological risk) (Figure 3B). In addition, Cd and Pb are the most harmful heavy metals, exceeding the geochemical background value in the average shale of the element. Therefore, the toxicity values for Cd and Pb needed further study in the middle Nile Delta, in order to assess their distribution on a larger scale, evaluate their potential impacts, and to explore a strategy for controlling these pollutants.

The Values of PERI were 413.04, 2534 and 4442.13 in the down-, middle- and upstream areas, respectively. The degree of sediment ecological damage for the three studied drains in the middle Nile Delta were at considerable to very high-risk levels. We noticed that the potential ecological risk increases in the up- and middlestream areas, compared to the downstream. The human activities and the small drains of the main studied streams play an important role in the environmental risks that threatens the region. Further, some brick factories located on the sides of the drains, as well as paint and leather factories, can affect the concentrations of heavy metals.

### 2.3. Heavy Metal Phytoremediation

#### 2.3.1. Heavy Metal Concentrations in Plants

The potential use of more developed plants as a sustainable and ecologically sound solution to the remediation of heavy metal contamination in the water, soil, sediment and sludges has attracted the attention of researchers and scientists worldwide [62]. The results for heavy metal concentrations in different organs (root and shoot) of the dominant plant species (*E. stagnina*, *P. australis*, and *T. domingensis*) growing along the banks of the studied drains are shown in Table S2. Heavy metal concentrations varied according to the plant species, plant organs, and locations. The ANOVA analysis revealed that most of the heavy metals (Fe, Mn, Cr, Co, Cd, and Ni) showed a significant variation (p < 0.05) between the studied species, except for Zn and Pb (Table S3). However, significant variations in the heavy metal contents between the roots and shoots of the studied plants were found. Significant variation, based on the plant organs, could be ascribed to the fact that roots are in direct contact with the sediment. Moreover, roots can accumulate more heavy metals due to the thick parenchyma cell of their cortex that embraces wide intercellular spaces [63]. In *P. australis*, Bonanno and Giudice [23] reported that the heavy metal concentrations in different organs were in the order of root > rhizome > leaf > stem. Cicero-Fernández et al. [24] reported that the roots of *P. australis* showed a higher accumulation capacity, compared to the shoots. Regarding the interaction between the plant species and the plant organs, Fe, Cu, Cr, Co, Cd, and Ni showed a significant variation, while Mn, Zn, and Pb did not show significant differences (Table S3).

The order of heavy metal accumulation was Fe > Mn > Ni >   Zn > Cu > Pb > Cd ≈ Co ≈ Cr for the root systems, and Mn > Fe > Zn > Pb > Cu > Ni > Cd > Co > Cr for the shoot systems of the studied emergent hydrophytes (Table S2). It can be clearly observed that the concentration of heavy metals in the studied emergent hydrophytes is higher in root tissue than in the shoot system. Regarding plant shoots, the highest levels of Fe and Zn concentrations were recorded in *P. australis*. Manganese was the highest in *E. stagnina*, while Cr, Co, Cd, Cu, Ni, and Pb were the highest in *T. domingensis*. Based on the heavy metal concentrations, the plant species can be ranked as follows: *P. australis > E. stagnina > T. domingensis*.

In the present study, all emergent hydrophytes had lower levels of Zn and Cu than the maximum normal value proposed by FAO/WHO [64], but they contained higher levels of other heavy metals (Cd, Ni, Mn, Co, Pb, Cr and Fe). Our results are in harmony with previous studies [18,65], showing higher levels of heavy metals in wild plants and crops.

#### 2.3.2. Assessment of Plants Ability for Heavy Metal Bioaccumulation

**Figure 4:**
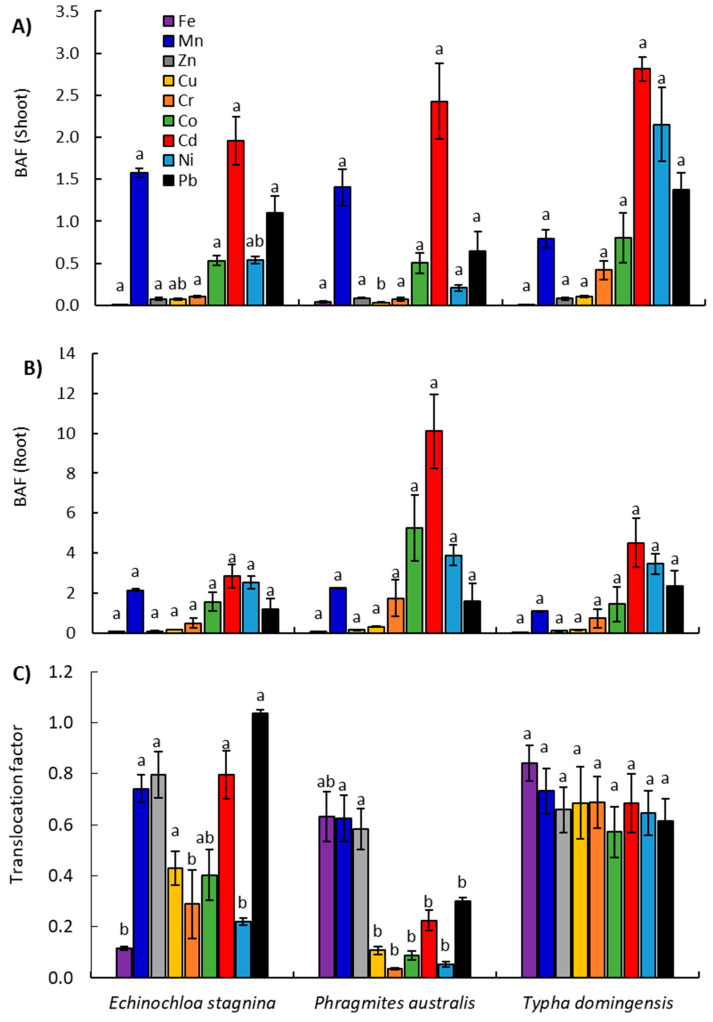
Bioaccumulation factor (BAF) of heavy metals in the shoots (**A**) and root (**B**), and the translocation factor (**C**) of the three studied emergent hydrophytes naturally growing along the studied drains. Different letters per each metal mean significant differences at values of *p* ≤ 0.05.

To evaluate the ability of the studied three emergent hydrophytes to extract and accumulate heavy metal in their tissues, the bioaccumulation factor (BAF) was calculated. According to BAF data, the uptake of heavy metals by plant samples followed the order Cd > Ni > Mn > Co > Pb > Cr > Cu > Zn > Fe for roots, while the order for shoots was Cd > Mn > Pb > Ni > Co > Cr > Cu > Zn > Fe. Generally, these observations were in harmony with previous studies [18,30]. The highest shoot BAF values were found in *T. domingensis* for most of the heavy metals, except Fe and Zn in *P. australis*, and Mn in *E. stagnina* (Figure 4A and Table S4). However, the lowest BAF of most of the elements was observed in *E. stagnina*.

On the other hand, the highest BAF value for all heavy metals in roots was found in *P. australis*, except for Pb, which accumulated more in the roots of *T. domingensis*. The lowest root BAF of Fe, Mn, Cu, and Co was observed in *E. stagnina*, while Zn, Cr, Cd, and Ni accumulated less in *T. domingensis*’s roots (Figure 4B and Table S4). Since the root is the organ responsible for absorption, the root BAF has been widely used to assess the translocation of heavy metals from the environment. Therefore, our data show that *P. australis* is the most effective accumulator for heavy metals, except for Pb, which is mainly an air-source pollutant. These results are in harmony with other studies using *P. australis* and *T. domingensis* as phytoremediators [22,24,25,26,27,29].

As mentioned above, the concentrations of Cd and Pb exceeded the limits in the studied drains. In this context, the BAF data of these elements show that *P. australis* roots have the best ability for accumulation of Cd, with a value of 10.11, while *T. domingensis* can accumulate Cd at a value of 5.24. On the other hand, *T. domingensis* and *P. australis* can accumulate Pb with BAF values of 2.37 and 1.61, respectively. The plant species with BAF > 1 are considered suitable for the phytoextraction of heavy metals [15], while the shoot could be considered an Mn, Cd and Pb accumulator.

Phytoremediation is an effective natural process whereby plants can accumulate numerous contaminants in different habitats. This process attracts the attention of researchers, scientists, and policymakers since it is a cost-effective, eco-friendly and promising technology [20]. *Phragmites australis* was reported as a good phytoremediator for many heavy metals in various habitats [22,24,25,26]. On the other hand, *T. domingensis* is characterized by a high morphological plasticity, enabling it to adapt to various environmental conditions and remove heavy metals with high efficiency [28]. *T. domingensis* was reported to accumulate heavy metals such as Cd, Cr, Cu, Mn, Ni, Pb and Zn [22].

In this context, the aquatic macrophytes are characterized by their easy propagation, fast growth rate, and immense biomass production within a short period [19]. Moreover, the emergent aquatic plants are usually more tolerant of, and efficient for the removal of, heavy metals compared to terrestrial plants [21].

The heavy metal distribution between the belowground and aboveground organs of *P. australis*, *E. stagnina* and *T. domingensis* was represented by the value of translocation factors (TF) (Figure 4C). According to the TF data, the highest values of Fe, Cu, Cr, Co and Ni (0.84, 0.69, 0.69, 0.57 and 0.65, respectively) were observed in *T. domingensis*, while those of Mn, Cd, Pb and Zn (0.74, 0.79, 0.80, and 1.04, respectively) were found in *E. stagnina* (Figure 4C and Table S4). In the present study, most plant species had a TF < 1 for Fe, Ni, Co and Cd, except the value for Pb being greater than one in the case of *E. stagnina*.

By comparing the results of TF, it is clear that heavy metals were preferentially localized in the roots, instead of being translocated into shoots, demonstrating a potential internal detoxification mechanism for heavy metals in the studied macrophytes [66]. These observations were in harmony with other studies [25,29]. The localization of the heavy metals in roots could be described as a mechanism of detoxification of the toxic metals, particularly with the cortex of the roots being thicker and having a larger intercellular space [22]. It is worth mentioning here that Mn showed a relatively consistent high TF in the three studied macrophytes, compared to other elements, where it attained values of 0.74, 0.73 and 0.62 for *E. stagnina*, *T. domingensis* and *P. australis*, respectively (Figure 4C). Since Mn is an essential element for photosynthesis and various enzymatic processes, the plant translocates this element from the belowground parts to leaves [29]. This finding is in harmony with other studies [22,26]. We can say that the accumulation of heavy metals in the present study is species- and organs-specific.

#### 2.3.3. Principal Component Analysis of the Plant Species and Heavy Metals

**Figure 5:**
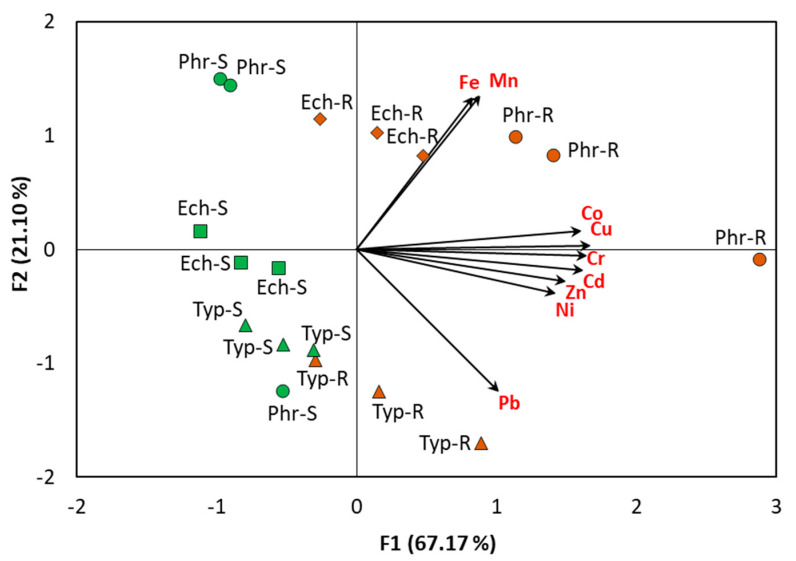
Principal component analysis of the heavy metals in the roots (R) and shoots (S) of *Echinochloa stagnina* (Ech), *Phragmites australis* (Phr) and *Typha domingensis* (Typ) based on the concentration of the nine heavy metals (Fe, Mn, Zn, Cu, Co, Cr, Ni, Cd and Pb).

The principal component analysis (PCA) of the roots and shoots of *E. stagnina*, *P. australis* and *T. domingensis*, based on the concentration of the nine heavy metals, revealed that the roots were positively correlated (p < 0.05) with all the analyzed heavy metals (Figure 5). In addition, *P. australis* roots showed more correlation with all elements except for Pb, where it showed less correlation. On the other hand, *T. domingensis* revealed greater correlation with Pb, while the roots of *E. stagnina* showed a positive correlation with Fe and Mn (Figure 5).

These data showed the low potential of heavy metal translocation from the belowground (roots) to the aboveground tissues (shoots), and revealed that metals bioaccumulated in the roots. Therefore, these plants can be considered metal excluders, and can be suitable for the phytoremediation of the sediments contaminated with heavy metals along the drains in the study areas, particularly Cd, Ni, Mn, Pb and Co [29].

## 3. Materials and Methods

### 3.1. Study Area

The Nile Delta is the most extensively used area in Egypt and is inhabited by 41% of the country’s population. It covers 2% of Egypt’s area and comprises ~63% of the total agriculture land (~29,600 km^2^). In addition, 40% of Egyptian industries are concentrated in the Nile Delta [8]. The Nile River is the primary source of water in the delta region, and 80% of its water is used in agriculture [67]. The northern lakes in the Nile Delta have a unique ecosystem, but they suffer from pollution due to the discharge of the wastewater from municipal, agricultural and industrial wastes into the waters [44].

In consequence, the Nile Delta has an extensive drainage system serving the agricultural sector. The main drains in the Nile Delta are El-Gharbia, Sabal, Elhoks, El Shakhlouba, Elkashaa, Bahr Tira, El-Serw, Hadous and Bahr El-Baqar. Burullus Lake occupies the central position along the Mediterranean coast of the Nile Delta (30°22`-31°35`N; 30°33`-31°08`E), with an area of about 460 km^2^. It is the second largest natural lake in Egypt after Lake Manzala. The main drains discharging in Burullus Lake are Elhoks (Drain 11), El Shakhlouba (Drain 9) and Drain 7 (Figure 1A), and they are the most severely polluted. The land use map of the study area was created based on Landsat 8 image acquired in August 2019 (Figure 1A) and was complemented with field observations (Figure 1B).

### 3.2. Sample Collection and Preparation

We selected three sites along each drain; these sites represent the downstream (near the Burullus Lake, and within the fish farms), middlestream (middle delta) and upstream (inside the delta) areas. The sites were selected to be far apart, with 20–30 km between them, and these drains extend along the whole area down the Burullus Lake, crossing the cultivated fields, urban areas, fish farms, and industrial areas (Figure 1A). During the spring of 2019, composite sediment samples (n = 3), at a depth of 15–30 cm, were collected from each site, air dried, sieved using a 2-mm sieve to remove gravel and debris, and stored in plastic bags for further chemical and physical analyses (total samples = 27 (3 drains × 3 sites per each drain × 3 replicas)). In addition, composite samples of three perennial common emergent macrophyte plants, namely, *E. stagnina*, *P. australis* and *T. domingensis*, were prepared. These plants were selected according to the field observations, where they naturally grow in all the sampled sites. Plants were collected from each studied drain in plastic bags. The plant samples were washed with tap water and distilled water to remove dust and then separated into shoots and roots. The samples were dried at 65 °C in an oven till complete dryness and ground into a powder with an electric grinder. Nomenclature and identification of the different plant species were carried out according to Boulos [68].

### 3.3. Samples Analyses

#### 3.3.1. Physicochemical Analysis of Sediment Samples

The sediment texture was determined via sieve method according to Piper [69], and organic matter (OM) was determined via the chromic acid titration method according to Walkley and Black [70]. Calcium carbonate was estimated via acid neutralization method according to Piper [69]. Soil suspension (1:5) was prepared and pH was determined using the pH meter (Model: YK-2001PH, Lutron, Malaysia), while electrical conductivity (EC) was measured by the conductivity meter (Model: CD-4306, Lutron, Malaysia).

#### 3.3.2. Chemical Analysis of Sediments and Plants

An acid extraction by microwave digestion (AR-MW) was followed by the chemical analysis of samples [71]. In sediment, 0.2 g of each sample was placed in a Teflon vessel with 12 mL of 37% HCl:70% HNO_3_ (3:1) mixture. For plant tissue, 0.2 g of each sample was placed in a Teflon vessel with 10 mL of aqua regia (70% HNO_3_:H_2_O_2_ (3:1) mixture). The vessels were heated in a microwave apparatus up to 180 °C within 5.5 min and remained at 180 °C for 9.5 min. The Inductivity Coupled Plasma optical emission spectrometer (Thermo Scientific™ iCAP™ 7000 Plus Series ICP-OES, USA) was used for the determination of nine metals (Fe, Mn, Zn, Cu, Co, Cr, Ni, Cd and Pb) of either sediment or plant tissue, with a standard calibration method. The wavelengths used in ICP-OES were selected according to ISO [72]. The accuracy of the elemental analysis was determined as precise, compared to the Certified Reference Materials (CRM), using stock standard solutions of Fe, Mn, Zn, Cu, Co, Cr, Ni, Cd and Pb (Merck, Darmstadt, Germany).

#### 3.3.3. Assessment of Sediment Heavy Metals Contamination

The pollution quantification for each metal along the drains was calculated through such indices as enrichment factor (E*f*), contamination factor (Cf), geoaccumulation index (Igeo), ecological risk factor (E*r*), degree of contamination (Dc), and potential ecological risk index (PERI) [55,59,61,73,74,75]. The calculation formulas for pollution indices and their class ranges are presented in Tables S5 and S6. 

#### 3.3.4. Assessment of Plants Ability for Heavy Metals Bioaccumulation

The metal bioaccumulation factor (BAF) of the roots or shoots of the three studied macrophytes was calculated as the ratio of the concentration of heavy metals in the root or shoots and its concentration in the sediment. The translocation factor (TF) represents the ratio between the concentration of the heavy metal in shoots and its concentration in the roots. Both BAF and TF were calculated according to Baker [76].

### 3.4. Statistical Analyses

To assess the variation among the upstream, middlestream and downstream sectors of the studied drains, the measured sediment variables, as well as heavy metal concentrations, were subjected to one-way ANOVA. The mean values of each parameter were separated based on Duncan’s test at the *p* ≤ 0.05 probability level, by the CoStat 6.3 program (CoHort Software, Monterey, CA, USA). In addition, the variations in heavy metal amounts in the collected plants, as well as their bioaccumulation factors and translocation factors, were tested by one-way ANOVA. Moreover, two-way ANOVA was performed to test the variations between the plant species, plant organs and their interactions. Pearson moment correlation analysis was performed to test the linear dependence among the analyzed heavy metals and sediment parameters.

A data matrix of the nine heavy metals’ (Fe, Mn, Zn, Cu, Co, Cr, Ni, Cd, and Pb) concentrations in the roots and shoots of *E. stagnina*, *P. australis* and *T. domingensis* from the studied sites was subjected to principal component analysis (PCA). The plot of the PCA of the plant samples and the projection of the heavy metal concentrations in different organs on the factor plane showed the similarities between the samples, and depicted the correlations between the original variable and the first two factors [77]. The PCA was performed via XLSTAT statistical computer software package, version 2018 (Addinsoft, New York, NY, USA).

## 4. Conclusions

The present data concerning various pollution indices (E*f*, Cf, Igeo, E*r*, Dc, and PERI) of the heavy metals in the sediment samples from three main drains in the middle Nile Delta revealed that the contamination by heavy metals increased from downstream to upstream, heading toward the Burullus Lake, for Cu, Ni and Co, while the reverse holds for Cr, Zn, and Cd. Moreover, Mn, Cr, Co, Cu, Ni, and Zn were recorded within the normal limits, while Cd and Pb showed a high to very high ecological risk index, which could be attributed to unremitting industrial activities. This high concentration of pollutants could be ascribed to the unremitting industrial human activities, such as brick factories located on the sides of the drains, as well as paint and leather factories. Unfortunately, farmers unofficially use agricultural drainage water, and as such these two pollutants can bioaccumulate in plants and animals, or bio-concentrate in the food chains, and cause serious problems for human health.

*P. australis* showed a potent accumulation of Cd, while *T. domingensis* attained the highest accumulation of Pb. Thus, these two emergent hydrophytes may be recommended as phytoremediators of these two hazardous heavy metals, which dominate the canal bank habitats along the drains in the middle Nile Delta region. Thus, this study showed the significance of using the emergent dominant hydrophytes of the drains in the middle Nile Delta region to stabilize the heavy metal contamination, and suggests phytoremediation as a promising, eco-friendly and cost-efficient method for the remediation of heavy metals in the polluted drains.

Therefore, strict laws and regulations must be taken into consideration by the policymaker against unmanaged industrial activities, particularly near the water streams in the Nile Delta. Moreover, the toxicity assessments of Cd and Pb need further study in the middle Nile Delta in order to assess their distribution on a broader scale, to evaluate their potential impacts on agriculture and human health, and to explore a strategy for controlling these pollutants.

## Figures and Tables

**Table 1:** Physicochemical characteristics of sediment samples collected from different sites (S1–S9) representing three sectors of drains moving toward Burullus Lake.

Parameter	Upstream				Middlestream				Downstream				*p-*Value
	S1	S4	S7	Mean ± SE	S2	S5	S8	Mean ± SE	S3	S6	S9	Mean ± SE	
pH	7.89	8.01	8.10	8.00 ^a^ ± 0.06	8.05	7.83	7.90	7.93 ^a^ ± 0.06	7.78	7.71	8.02	7.84 ^a^ ± 0.09	0.36 ^ns^
EC (dS/m)	2.14	1.89	1.91	1.98 ^c^ ± 0.08	2.88	2.46	3.15	2.83 ^b^ ± 0.20	3.55	3.22	3.72	3.50 ^a^ ± 0.15	0.001 **
OM (%)	1.32	1.37	1.51	1.40 ^b^ ± 0.06	1.65	2.17	2.08	1.97 ^ab^ ± 0.16	1.98	2.88	2.92	2.59 ^a^ ± 0.31	0.017 *
CaCO_3_ (%)	2.95	2.80	1.79	2.51 ^a^ ± 0.36	2.43	2.28	2.42	2.38 ^a^ ± 0.05	2.63	2.68	2.88	2.73 ^a^ ± 0.08	0.54 ^ns^
Sand (%)	81.10	70.97	78.73	76.93 ^b^ ± 3.06	85.32	77.78	83.29	82.13 ^ab^ ± 2.25	89.75	86.89	90.12	88.92 ^a^ ± 1.02	0.027 *
Silt (%)	13.24	17.58	12.52	14.45 ^a^ ± 1.58	11.57	12.58	9.67	11.27 ^ab^ ± 0.85	8.27	9.57	7.22	8.35 ^b^ ± 0.68	0.023 *
Clay (%)	5.66	11.45	8.75	8.62 ^a^ ± 1.67	3.11	9.64	7.04	6.60 ^ab^ ± 1.90	1.98	3.54	2.66	2.73 ^a^ ± 0.45	0.076 ^ns^

SE: standard error; EC: Electrical conductivity; OM: Soil organic matter; different superscript letters within each row mean values significant at *p* ≤ 0.05; **: significant at *p* ≤ 0.01, *: significant at *p* ≤ 0.05, ^ns^: non-significance.

**Table 2:** Heavy metal concentrations (mg/kg) in sediment samples from different sites (S1–S9) representing the three main drains of the Burullus Lake.

Locations	Site	Fe	Mn	Pb	Cr	Zn	Cu	Ni	Co	Cd
Upstream	S1	45,624.00	776.70	441.20	397.90	51.20	7.33	5.22	9.98	54.68
	S4	61,201.00	1350.30	599.60	531.20	18.40	5.16	2.08	33.37	40.95
	S7	32,079.00	776.80	772.20	401.40	58.00	12.78	3.66	50.86	29.76
	Mean	46,301.33 ^a^	967.93 ^a^	604.33 ^b^	443.50 ^a^	42.53 ^a^	8.42 ^a^	3.65 ^a^	31.40 ^a^	41.80 ^a^
	± SE	8413.62	191.18	95.58	43.86	12.23	2.27	0.91	11.84	7.21
Middlestream	S2	40,742.50	782.13	467.48	232.55	38.75	10.40	4.53	19.48	30.90
	S5	71,617.00	801.78	1073.20	287.39	19.50	8.47	5.21	28.14	22.47
	S8	41,711.00	781.00	723.10	218.48	56.75	12.13	5.11	48.14	16.50
	Mean	51,356.83 ^a^	788.30 ^ab^	754.59 ^a^	246.14 ^b^	38.33 ^a^	10.33 ^a^	4.95 ^a^	31.92 ^a^	23.29 ^b^
	± SE	10,133.94	6.75	175.56	21.02	10.76	1.06	0.21	8.49	4.18
Downstream	S3	30,861.00	1123.05	158.25	67.19	26.30	13.46	3.84	28.98	0.71
	S6	72,033.00	1003.95	796.10	43.58	20.60	11.78	8.33	22.90	3.99
	S9	51,343.00	789.80	669.40	35.55	55.50	11.48	6.56	45.41	3.24
	Mean	51,412.33 ^a^	972.27 ^a^	541.25 ^a^	48.77 ^c^	34.13 ^a^	12.24 ^a^	6.24 ^a^	32.43 ^a^	2.65 ^c^
	± SE	11,885.38	97.50	194.96	9.50	10.81	0.62	1.31	6.72	0.99
*p*−value		0.92 ^ns^	0.0450 *	0.34 ^ns^	0.0002 ***	0.87 ^ns^	0.27 ^ns^	0.22 ^ns^	0.99 ^ns^	0.0037 **
**Permissible limits worldwide**										
EU (2002)		-	-	300	150	300	140	75	11.6	3
CSQGD (2007)		-	-	70	64	-	-	50	40	1.4
US EPA (1999)		-	550	19	54	60	25	19	9.1	0.01–41
Average Shale		47,200	850	20	90	95	45	68	19	0.3
Toxic response factor		-	1	5	2	1	5	5	5	30

SE: standard error; different superscript letters within each column mean values significant at *p* ≤ 0.05; EU: European Union Standard (2002); CSQGD: Canadian soil quality guidelines for the protection of environmental and human health document (2007); US EPA (United States Environmental Protection Agency) (1999). ***: significant at *p* ≤ 0.001, **: significant at *p* ≤ 0.01, *: significant at *p* ≤ 0.05, ^ns^: non-significance.

## Supplementary Materials

The following are available online at https://www.mdpi.com/2223-7747/9/7/910/s1, Table S1: Pearson correlation matrix various sediment parameters and heavy metals from the three studied drains in Nile Delta, Table S2: Microelement concentrations (mg/kg) in roots and shoots of three studied emergent hydrophytes naturally growing along studied drains, Table S3: Results of analysis of variance of various studied heavy metals in either roots or shoots of *Echinochloa stagnina*, *Phragmites australis*, and *Typha domingensis* collected from the three studied drains in Nile Delta. Table S4: Comparison among the three plant species (*Echinochloa stagnina*, *Phragmites australis*, and *Typha domingensis*) based on the bioaccumulation factors of root and shoot as well as the translocation factor. Table S5: Various pollution indices formulas used in the present study, Table S6: Classes of used indices for metals in the present study.
