# Supplementary material for: Ecological Risk Assessment of Heavy Metals along Three Main Drains in Nile Delta and Potential Phytoremediation by Macrophyte Plants
Source: Plants (Basel). 2020 Jul 18;9(7):910. doi: 10.3390/plants9070910 (PMC7412368; doi:10.3390/plants9070910)
Supplement: Supplementary file 1 [file plants-09-00910-s001.pdf]

# Ecological Risk Assessment of Heavy Metals along Three Main Drains in Nile Delta, and Potential Phytoremediation by Macrophyte Plants

**Yasser A. El-Amier[1], Giuliano Bonanomi[2], Saud L. Al-Rowaily[3] and Ahmed M. Abd-ElGawad[1,3]***

[1] Department of Botany, Faculty of Science, Mansoura University, Mansoura 35516, Egypt; yasran@mans.edu.eg, dgawa84@mans.edu.eg

[2] Department of Agriculture, University of Naples Federico II, 80055, Portici, Naples, Italy; giuliano.bonanomi@unina.it

[3] Plant Production Department, College of Food & Agriculture Sciences, King Saud University, P.O. Box 2460 Riyadh 11451, Saudi Arabia; aibrahim2@ksu.edu.sa, srowaily@ksu.edu.sa

* Correspondence: aibrahim2@ksu.edu.sa, Tel.: +966562680864 (A.M.A-E.)

## Supplementary Materials

**Table S1.** Pearson correlation matrix various sediment parameters and heavy metals from the three studied drains in Nile Delta.

| Variables | Fe | Mn | Pb | Cr | Zn | Cu | Ni | Co | Cd | pH | EC | OM | $CaCO_3$ | Sand | Silt |
|---|---|---|---|---|---|---|---|---|---|---|---|---|---|---|---|
| Mn | 0.22 | | | | | | | | | | | | | | |
| Pb | 0.65 | -0.32 | | | | | | | | | | | | | |
| Cr | -0.01 | 0.18 | 0.12 | | | | | | | | | | | | |
| Zn | -0.62 | **-0.69** | -0.06 | 0.01 | | | | | | | | | | | |
| Cu | -0.49 | -0.34 | -0.14 | **-0.71** | 0.36 | | | | | | | | | | |
| Ni | 0.43 | -0.42 | 0.32 | **-0.71** | 0.06 | 0.35 | | | | | | | | | |
| Co | -0.26 | -0.09 | 0.31 | -0.05 | 0.44 | 0.43 | -0.17 | | | | | | | | |
| Cd | -0.05 | -0.05 | -0.03 | **0.89** | 0.13 | **-0.74** | -0.48 | -0.38 | | | | | | | |
| pH | -0.42 | -0.21 | 0.00 | 0.49 | 0.54 | -0.14 | -0.51 | 0.42 | 0.42 | | | | | | |
| EC | -0.07 | -0.11 | -0.24 | **-0.95** | 0.05 | 0.63 | 0.56 | 0.11 | **-0.85** | -0.39 | | | | | |
| OM | 0.43 | -0.14 | 0.33 | **-0.85** | -0.09 | 0.46 | **0.81** | 0.21 | **-0.84** | -0.44 | **0.78** | | | | |
| $CaCO_3$ | 0.28 | 0.35 | -0.41 | -0.20 | -0.20 | -0.41 | 0.24 | -0.48 | 0.02 | -0.32 | 0.32 | 0.20 | | | |
| Sand | -0.29 | -0.32 | -0.37 | **-0.93** | 0.24 | **0.74** | 0.61 | -0.03 | **-0.71** | -0.31 | **0.90** | 0.64 | 0.22 | | |
| Silt | 0.24 | 0.40 | 0.15 | **0.94** | -0.31 | **-0.83** | -0.63 | -0.22 | **0.82** | 0.32 | **-0.91** | **-0.74** | -0.06 | **-0.95** | |
| Clay | 0.30 | 0.22 | 0.54 | **0.83** | -0.16 | -0.60 | -0.54 | 0.25 | 0.55 | 0.28 | **-0.81** | -0.49 | -0.34 | **-0.96** | **0.81** |

Values in bold are significance at $p \leq 0.05$. OM: soil organic matter, EC: electric conductivity.

**Table S2.** Microelement concentrations (mg kg$^{-1}$) in roots and shoots of three studied emergent hydrophytes naturally growing along studied drains.

| Plant species | Plant part | Drain | Metals | | | | | | | | |
|---|---|---|---|---|---|---|---|---|---|---|---|
| | | | Fe | Mn | Zn | Cu | Cr | Co | Cd | Ni | Pb |
| *Echinochloa stagnina* | Root | (Drain 11) | 2585.87 | 1564.19 | 50.28 | 33.17 | 11.76 | 10.48 | 8.66 | 63.13 | 16.65 |
| | | (Drain 9) | 2761.67 | 1739.99 | 78.10 | 48.98 | 18.72 | 20.94 | 20.46 | 78.94 | 32.45 |
| | | (Drain 7) | 2755.20 | 1733.52 | 71.63 | 42.50 | 12.25 | 14.47 | 13.99 | 72.47 | 25.98 |
| | | Mean | 2700.91 | 1679.23 | 66.67 | 41.55 | 14.24 | 15.30 | 14.37 | 71.51 | 25.03 |
| | | ±SE | 57.55 | 57.55 | 8.41 | 4.59 | 2.24 | 3.05 | 3.41 | 4.59 | 4.59 |
| | Shoot | (Drain 11) | 326.40 | 1252.56 | 55.46 | 16.30 | 1.93 | 4.21 | 9.68 | 13.18 | 25.92 |
| | | (Drain 9) | 275.09 | 1098.84 | 34.44 | 14.66 | 2.74 | 3.76 | 4.58 | 16.07 | 11.76 |
| | | (Drain 7) | 331.29 | 1372.08 | 60.36 | 21.19 | 6.82 | 9.10 | 14.58 | 18.07 | 30.81 |
| | | Mean | 310.93 | 1241.16 | 50.09 | 17.38 | 3.83 | 5.69 | 9.61 | 15.77 | 22.83 |
| | | SE | 17.97 | 79.08 | 7.95 | 1.96 | 1.51 | 1.71 | 2.89 | 1.42 | 5.71 |
| *Phragmites australis* | Root | (Drain 11) | 2778.39 | 1756.71 | 94.81 | 65.69 | 35.44 | 37.66 | 37.18 | 95.65 | 21.16 |
| | | (Drain 9) | 2813.09 | 1791.41 | 129.52 | 100.39 | 70.14 | 72.36 | 71.88 | 130.36 | 55.87 |
| | | (Drain 7) | 2783.70 | 1762.02 | 100.12 | 71.00 | 40.75 | 42.97 | 42.49 | 100.96 | 26.47 |
| | | Mean | 2791.73 | 1770.05 | 108.15 | 79.03 | 48.78 | 51.00 | 50.52 | 108.99 | 34.50 |
| | | ±SE | 10.79 | 10.79 | 10.79 | 10.79 | 10.79 | 10.79 | 10.79 | 10.79 | 10.79 |
| | Shoot | (Drain 11) | 2457.55 | 1435.87 | 47.98 | 5.51 | 1.16 | 2.17 | 6.50 | 3.30 | 2.33 |
| | | (Drain 9) | 350.76 | 430.08 | 96.46 | 13.87 | 3.01 | 8.27 | 22.01 | 9.61 | 35.74 |
| | | (Drain 7) | 2459.35 | 1437.67 | 49.78 | 7.31 | 1.38 | 3.97 | 8.30 | 5.10 | 4.13 |
| | | Mean | 1755.89 | 1101.21 | 64.74 | 8.90 | 1.85 | 4.80 | 12.27 | 6.00 | 14.07 |
| | | ±SE | 702.56 | 335.56 | 15.87 | 2.54 | 0.58 | 1.81 | 4.90 | 1.88 | 10.85 |
| *Typha domingensis* | Root | (Drain 11) | 641.01 | 841.58 | 69.38 | 29.15 | 8.55 | 2.65 | 11.86 | 86.75 | 39.43 |
| | | (Drain 9) | 664.64 | 865.22 | 93.02 | 52.79 | 32.19 | 26.29 | 35.50 | 110.38 | 63.07 |
| | | (Drain 7) | 650.13 | 850.70 | 78.50 | 38.27 | 17.67 | 11.77 | 20.98 | 95.87 | 48.55 |
| | | Mean | 651.93 | 852.50 | 80.30 | 40.07 | 19.47 | 13.57 | 22.78 | 97.67 | 50.35 |
| | | ±SE | 6.88 | 6.88 | 6.88 | 6.88 | 6.88 | 6.88 | 6.88 | 6.88 | 6.88 |
| | Shoot | (Drain 11) | 555.08 | 728.77 | 60.08 | 25.25 | 7.40 | 2.30 | 10.27 | 75.12 | 31.43 |
| | | (Drain 9) | 473.71 | 303.43 | 26.45 | 21.59 | 16.55 | 16.92 | 16.84 | 26.59 | 18.84 |
| | | (Drain 7) | 614.52 | 831.84 | 64.80 | 29.96 | 12.12 | 2.40 | 14.99 | 79.84 | 36.14 |
| | | Mean | 547.77 | 621.35 | 50.44 | 25.60 | 12.02 | 7.21 | 14.03 | 60.52 | 28.80 |
| | | ±SE | 40.81 | 161.72 | 12.07 | 2.42 | 2.64 | 4.86 | 1.96 | 17.02 | 5.16 |

**Table S3.** Results of analysis of variance of various studied heavy metals in either roots or shoots of *Echinochloa stagnina*, *Phragmites australis*, and *Typha domingensis* collected from the three studied drains in Nile Delta.

| Element | Effect | SS | df | MS | *F* | *P* |
|---|---|---|---|---|---|---|
| Fe | Species | 8425504.60 | 2 | 4212752.3 | 16.88 | **0.0003*** ** |
| | Organ | 6230391.17 | 1 | 6230391.2 | 24.97 | **0.0003*** ** |
| | Species × Organ | 3963382.91 | 2 | 1981691.5 | 7.94 | **0.0064** ** |
| Mn | Species | 2023828.70 | 2 | 1011914.3 | 13.62 | **0.0008*** ** |
| | Organ | 895211.20 | 1 | 895211.2 | 12.06 | **0.0046** ** |
| | Species × Organ | 143819.38 | 2 | 71909.69 | 0.97 | 0.4074 |
| Zn | Species | 2561.46 | 2 | 1280.73 | 3.68 | 0.0566$^{ns}$ |
| | Organ | 4036.51 | 1 | 4036.51 | 11.61 | **0.0052** ** |
| | Species × Organ | 539.77 | 2 | 269.89 | 0.78 | 0.4819$^{ns}$ |
| Cu | Species | 690.51 | 2 | 345.25 | 3.43 | 0.0662$^{ns}$ |
| | Organ | 5915.09 | 1 | 5915.09 | 58.84 | **<0.0001*** ** |
| | Species × Organ | 2652.34 | 2 | 1326.17 | 13.19 | **0.0009*** ** |
| Cr | Species | 802.95 | 2 | 401.48 | 4.50 | **0.0348* ** |
| | Organ | 2098.66 | 1 | 2098.66 | 23.52 | **0.0004*** ** |
| | Species × Organ | 1450.35 | 2 | 725.17 | 8.13 | **0.0059** ** |
| Co | Species | 1219.32 | 2 | 609.66 | 6.01 | **0.0156* ** |
| | Organ | 1932.14 | 1 | 1932.14 | 19.04 | **0.0009*** ** |
| | Species × Organ | 1467.77 | 2 | 733.88 | 7.23 | **0.0087** ** |
| Cd | Species | 1172.46 | 2 | 586.23 | 5.54 | **0.0197* ** |
| | Organ | 1339.03 | 1 | 1339.03 | 12.66 | **0.0039** ** |
| | Species × Organ | 1003.88 | 2 | 501.94 | 4.74 | **0.0303* ** |
| Ni | Species | 3829.69 | 2 | 1914.84 | 7.98 | **0.0063** ** |
| | Organ | 19183.83 | 1 | 19183.83 | 79.92 | **<0.0001*** ** |
| | Species × Organ | 3456.15 | 2 | 1728.07 | 7.20 | **0.0088** ** |
| Pb | Species | 957.76 | 2 | 478.88 | 2.65 | 0.1117$^{ns}$ |
| | Organ | 975.79 | 1 | 975.79 | 5.39 | **0.0386* ** |
| | Species × Organ | 354.12 | 2 | 177.06 | 0.98 | 0.4040$^{ns}$ |

Notes: Tested effects included plant species and plant organs. For each tested effect, sum of squares (SS), degrees of freedom (df) mean squares (MS) and Duncan's test results (*F* and associated *s* value [significant values in boldface type]) are shown. Significance level fixed at *p*-values < 0.05. $^{***}$: significant at $p \leq 0.001$, $^{**}$: significant at $p \leq 0.01$, $^{*}$: significant at $p \leq 0.05$, $^{ns}$: non-significant.

**Table S4.** Comparison among the three plant species (*Echinochloa stagnina*, *Phragmites australis*, and *Typha domingensis*) based on the bioaccumulation factors of root and shoot as well as the translocation factor.

| **Factor** | | **Fe** | **Mn** | **Zn** | **Cu** | **Cr** | **Co** | **Cd** | **Ni** | **Pb** |
|---|---|---|---|---|---|---|---|---|---|---|
| ***Root bioaccumulation factor (BAF)*** | | | | | | | | | | |
| *Echinochloa stagnina* | Mean | 0.058 $^{A}$ | 2.130 $^{A}$ | 0.093 $^{A}$ | 0.169 $^{B}$ | 0.493 $^{A}$ | 1.558 $^{A}$ | 2.860 $^{B}$ | 2.517 $^{A}$ | 1.199 $^{A}$ |
| | ±SE | 0.005 | 0.038 | 0.006 | 0.009 | 0.136 | 0.266 | 0.338 | 0.301 | 0.180 |
| *Phragmites australis* | Mean | 0.060 $^{A}$ | 2.245 $^{A}$ | 0.154 $^{A}$ | 0.319 $^{A}$ | 1.743 $^{A}$ | 5.236 $^{A}$ | 10.109 $^{A}$ | 3.880 $^{A}$ | 1.609 $^{A}$ |
| | ±SE | 0.005 | 0.004 | 0.014 | 0.011 | 0.536 | 0.955 | 1.067 | 0.517 | 0.292 |
| *Typha domingensis* | Mean | 0.014 $^{B}$ | 1.081 $^{B}$ | 0.115 $^{A}$ | 0.161 $^{B}$ | 0.728 $^{A}$ | 1.443 $^{A}$ | 4.514 $^{B}$ | 3.456 $^{A}$ | 2.373 $^{A}$ |
| | ±SE | 0.001 | 0.002 | 0.010 | 0.011 | 0.267 | 0.494 | 0.710 | 0.432 | 0.290 |
| F-value | | 12.164 | 275.197 | 2.701 | 25.232 | 1.171 | 3.796 | 8.215 | 0.894 | 1.759 |
| P-value | | 0.0077 $^{**}$ | <0.0001 $^{***}$ | 0.1457 $^{ns}$ | 0.0012 $^{**}$ | 0.3721 $^{ns}$ | 0.0860 $^{ns}$ | 0.0191 $^{*}$ | 0.4573 $^{ns}$ | 0.2505 $^{ns}$ |
| ***Root bioaccumulation factor (BAF)*** | | | | | | | | | | |
| *Echinochloa stagnina* | Mean | 0.007 $^{A}$ | 1.576 $^{A}$ | 0.078 $^{A}$ | 0.073 $^{AB}$ | 0.103 $^{A}$ | 0.533 $^{A}$ | 1.957 $^{A}$ | 0.541 $^{A}$ | 1.097 $^{A}$ |
| | ±SE | 0.001 | 0.065 | 0.015 | 0.008 | 0.016 | 0.063 | 0.333 | 0.051 | 0.231 |
| *Phragmites australis* | Mean | 0.042 $^{A}$ | 1.404 $^{A}$ | 0.087 $^{A}$ | 0.035 $^{B}$ | 0.070 $^{A}$ | 0.504 $^{A}$ | 2.429 $^{A}$ | 0.206 $^{A}$ | 0.641 $^{A}$ |
| | ±SE | 0.011 | 0.251 | 0.006 | 0.004 | 0.025 | 0.138 | 0.520 | 0.041 | 0.276 |
| *Typha domingensis* | Mean | 0.012 $^{A}$ | 0.792 $^{A}$ | 0.081 $^{A}$ | 0.107 $^{A}$ | 0.418 $^{A}$ | 0.806 $^{A}$ | 2.812 $^{A}$ | 2.153 $^{A}$ | 1.374 $^{A}$ |
| | ±SE | 0.001 | 0.121 | 0.017 | 0.010 | 0.124 | 0.344 | 0.165 | 0.506 | 0.239 |
| F-value | | 3.115 | 2.081 | 0.039 | 7.037 | 2.255 | 0.196 | 0.449 | 4.169 | 0.734 |
| P-value | | 0.1181 $^{ns}$ | 0.2058 $^{ns}$ | 0.9620 $^{ns}$ | 0.0267 $^{*}$ | 0.1860 $^{ns}$ | 0.8273 $^{ns}$ | 0.6582 $^{ns}$ | 0.0733 $^{ns}$ | 0.5185 $^{ns}$ |
| ***Translocation factor (TF)*** | | | | | | | | | | |
| *Echinochloa stagnina* | Mean | 0.115 $^{B}$ | 0.741 $^{A}$ | 0.796 $^{A}$ | 0.430 $^{AB}$ | 0.289 $^{A}$ | 0.403 $^{B}$ | 0.795 $^{A}$ | 0.221 $^{B}$ | 1.035 $^{A}$ |
| | ±SE | 0.014 | 0.095 | 0.334 | 0.113 | 0.232 | 0.225 | 0.496 | 0.025 | 0.611 |
| *Phragmites australis* | Mean | 0.631 $^{AB}$ | 0.624 $^{A}$ | 0.583 $^{A}$ | 0.108 $^{B}$ | 0.037 $^{B}$ | 0.088 $^{A}$ | 0.225 $^{A}$ | 0.053 $^{B}$ | 0.302 $^{A}$ |
| | ±SE | 0.438 | 0.333 | 0.140 | 0.028 | 0.006 | 0.029 | 0.071 | 0.020 | 0.293 |
| *Typha domingensis* | Mean | 0.841 $^{A}$ | 0.731 $^{A}$ | 0.659 $^{A}$ | 0.686 $^{A}$ | 0.689 $^{A}$ | 0.572 $^{A}$ | 0.685 $^{A}$ | 0.647 $^{A}$ | 0.613 $^{A}$ |
| | ±SE | 0.118 | 0.334 | 0.325 | 0.244 | 0.176 | 0.338 | 0.197 | 0.352 | 0.274 |
| F-value | | 6.0846 | 0.1630 | 0.4431 | 10.3529 | 11.4825 | 3.2809 | 2.8323 | 6.7596 | 2.2782 |
| P-value | | 0.0360 $^{*}$ | 0.8532 $^{ns}$ | 0.6615 $^{ns}$ | 0.0113 $^{*}$ | 0.0089 $^{**}$ | 0.1090 $^{ns}$ | 0.1361 $^{ns}$ | 0.0290 $^{*}$ | 0.1836 $^{ns}$ |

Different superscript letters within each element and factor mean values significance. Significance level fixed at *p*-values < 0.05. $^{***}$: significant at $p \leq 0.001$, $^{**}$: significant at $p \leq 0.01$, $^{*}$: significant at $p \leq 0.05$, $^{ns}$: non-significant.

**Table S5.** Various pollution indices formulas used in the present study.

| Index | Formula | References |
|---|---|---|
| ***Single indices of pollution*** | | |
| Enrichment factor (E*f*) | $\mathbf{EF} = \left(\frac{\mathbf{C_{sample}}}{\mathbf{Fe_{sample}}}\right) / \left(\frac{\mathbf{C_{ref}}}{\mathbf{Fe_{ref}}}\right)$ | Franco-Uria et al. (2009) |
| Contamination factor (C*f*) | $\mathbf{CF} = \boldsymbol{C_{sample}} \;/\; \boldsymbol{C_{ref}}$ | Hakanson (1980) |
| Geoaccumulation index (I*geo*) | $\mathbf{I}\boldsymbol{geo} = \mathbf{Log2}\left(\frac{\mathbf{C_{sample}}}{\mathbf{1.5Bn}}\right)$ | Muller (1969); Lu and Bai (2010) |
| Ecological risk factor (E*r*) | $\mathbf{Er} = \mathbf{Ti} * \mathbf{Cf}$ | Hakanson (1980) |
| ***Total complex indices (include integrated indices and indices of ecological risk)*** | | |
| Degree of contamination (D*c*) | $\mathbf{Dc} = \sum_{\mathbf{i=1}}^{\mathbf{n}} \mathbf{CFi}$ | Hakanson (1980); Caeiro et al. (2005) |
| Potential ecological risk index (PERI) | $\mathbf{PERI} = \sum_{\mathbf{i=1}}^{\mathbf{n}} \mathbf{ER}$ | Kowalska et al. (2016) |
| Bioaccumulation factor (BAF) | $\mathbf{BAF_{shoot/root}} = \frac{\mathbf{Metal_{shoot/root}}}{\mathbf{Metal_{soil}}}$ | Baker (1981) |
| Translocation factor (TF) | $\mathbf{TF} = \frac{\mathbf{Metal_{shoot}}}{\mathbf{Metal_{root}}}$ | Baker (1981) |

**Abbreviation:** $C_{sample}$: metal concentration in soil analyzed sample; Fe $_{sample}$: concentration of the reference metal in soil analyzed sample; $C_{ref}$ : (background) metal concentration in the reference environment; $Fe_{ref}$ (background), reference metal concentration in the reference environment; Bn: the geochemical background value in average shale of element *n*; 1.5: the background matrix correction due to terrigenous effects; Ti: the toxic-response factor for a given substance; Cf: the contamination factor.

**Table S6.** Classes of used indices for metals in the present study.

| Index | Value | Soil quality | Ecological risk |
|---|---|---|---|
| E*f* | EF < 2 = natural, EF > 2 = anthropogenic | | |
| | Ef < 1 | Depletion or no enrichment | |
| | Ef < 2 | Minor enrichment | |
| | Ef = 2-5 | Moderate enrichment | |
| | Ef = 5-10 | Moderately severe enrichment | |
| | Ef = 10-25 | Severe enrichment | |
| | Ef = 25-50 | Very severe enrichment | |
| | Ef > 50 | Extremely severe enrichment | |
| C*f* | CF < 1 | Low contamination factor | |
| | 1 ≤ CF ≤ 3 | Moderate contamination factor | |
| | 3 ≤ CF ≤ 6 | Considerable contamination factor | |
| | 6 ≤ CF | Very high contamination factor | |
| D*c* | DC < 8 | Low DC | |
| | 8 ≤ Dc < 16 | Moderate DC | |
| | 16 ≤ Dc < 32 | Considerable DC | |
| | Dc > 32 | Very high | |
| I*geo* | I*geo* ≤ 0 | Uncontaminated | |
| | 0 < I*geo* < 1 | Uncontaminated to moderately contaminated | |
| | 1 < I*geo* < 2 | Moderately to heavily contaminated | |
| | 2 < I*geo* < 3 | Moderately to strongly contaminated | |
| | 3 < I*geo* < 4 | Strongly contaminated | |
| | 4 < I*geo* < 5 | Strongly to extremely contaminated | |
| | I*geo* > 5 | Extremely high contaminated | |
| E*r* | Er < 40 | | Low ecological risk |
| | 40 ≤ Er < 80 | | Moderate ecological risk |
| | 80 ≤ Er < 160 | | Considerable ecological risk |
| | 160 ≤ Er < 320 | | High ecological risk |
| | Er ≥ 320 | | Very high ecological risk |
| PERI | PERI < 150 | | Low risk |
| | 150 ≤ PERI < 300 | | Moderate |
| | 300 ≤ PERI < 600 | | Considerable |
| | PERI ≥ 600 | | Very high. |

Abbrivation: Enrichment factor (EF), Contamination factor (CF), Degree of contamination (Dc), Geoaccumulation index (I*geo*), Ecological risk factor (Er) and Potential ecological risk index (PERI)
